# Curcumin induces apoptosis in osteosarcoma cells by regulating the glycolytic pathway via the MAPK axis: a mechanistic study

**DOI:** 10.1515/biol-2025-1296

**Published:** 2026-03-10

**Authors:** Yi Wang, Yuxi Zhang, Yuhan Zhang, Jierui Zhao, Jinlian Liao, Jingwen Luo, Min Chen, Peng Peng

**Affiliations:** Faculty of Chinese Medicine and State Key Laboratory of Quality Research in Chinese Medicines, Macau University of Science and Technology, Macau, Macau SAR, 999078, China; The Islands Healthcare Complex – Macao Medical Center of Peking Union Medical College Hospital, Macao, Macao SAR, 999078, China; Changchun University of Chinese Medicine, Changchun, Jilin 130012, China

**Keywords:** curcumin, osteosarcoma cells, glycolysis, apoptosis, P38/JNK

## Abstract

This study investigated the mechanism by which curcumin induces apoptosis through glycolytic regulation in osteosarcoma cells (U2OS, MG63) via the MAPK axis. Network pharmacology, protein-protein interaction analysis, and molecular docking were integrated to identify core targets and validate the central role of the MAPK pathway. *In vitro*, curcumin dose-dependently inhibited cell proliferation (IC50: 32.6 μmol/L in MG63, 37.3 μmol/L in U2OS) and migration, while promoting apoptosis as confirmed by CCK-8, wound healing, flow cytometry, and TUNEL assays. Western blot analysis demonstrated that curcumin significantly upregulated the expression of pro-apoptotic proteins Bax and Cleaved-PARP, as well as phosphorylated P38 and JNK (P-P38, P-JNK), while downregulating the glycolytic enzymes HK2 and PKM2 and the anti-apoptotic protein Bcl-2 (all *P* < 0.05). Total JNK and P38 levels remained unchanged. The TUNEL assay further confirmed concentration-dependent enhancement of apoptosis, with a marked increase in TUNEL-positive cells at 40 μmol/L. Combined bioinformatic and experimental evidence indicates that curcumin inhibits glycolysis and promotes osteosarcoma cell apoptosis primarily through activation of the JNK/P38 MAPK signaling branch.

## 1 Introduction

Osteosarcoma (OS) is the most common primary malignant bone tumor, predominantly affecting children and adolescents. It originates from mesenchymal stem cells and is characterized by osteoid formation. Osteosarcoma predominantly affects the metaphysis of long bones in the extremities, such as the distal femur, proximal tibia, proximal femur, and proximal humerus. Approximately 60 % of patients are reported to be between 10 and 20 years old, making osteosarcoma the second leading cause of death in this age group. The annual incidence of osteosarcoma is 3–5 cases per million population. Despite its low incidence, osteosarcoma carries a high rate of disability and mortality [1]. The 5-year relative survival rate for patients with localized limb tumors is 70 %, but survival drops significantly below 30 % for those with distant metastases. Despite advances in treating localized disease, primarily through surgical resection or stereotactic radiotherapy, controlling metastatic spread remains a formidable challenge, with survival rates showing no improvement over the past two decades [2]. In clinical settings, metastatic progression to lung tissue and subsequent patient recurrence remain the primary cause of death in OS patients. Current treatment for OS patients typically involves surgery combined with adjuvant and neoadjuvant chemotherapy. Up to 25 % of patients present with evidence of metastatic disease at diagnosis, with lung metastases being the most common [3]. The survival rate for metastatic OS patients is low, with less than 5 years [4].

Curcumin (Cur), derived from turmeric, is a diarylheptane compound and a traditional Chinese medicine used to promote blood circulation and remove blood stasis. Long employed in China for treating pain and inflammation, it has been reported to possess antioxidant, anti-inflammatory, immunomodulatory, and anticancer effects [5], 6]. Notably, its anticancer effects have been extensively studied, revealing its ability to inhibit cancer cell proliferation, migration, and metastasis by regulating signaling pathways, induce apoptosis, impede tumor angiogenesis, and reverse multidrug resistance [7]. Liu et al. [8] found curcumin induces mesenchymal-epithelial transition (MET) in colorectal cancer (CRC) cells via a miR-34a-dependent mechanism and inhibits lung metastasis formation in mice. Mayo et al. [9] demonstrated curcumin prevents cancer initiation and progression by suppressing signaling pathways including COX-2, CD-31, VEGF, and IL-8, while regulating the expression of pro-tumorigenic proteins. Ghasemi et al. [10] demonstrated that curcumin inhibits invasion and proliferation of cervical cancer cells by suppressing NF-κB and Wnt/β-catenin signaling pathways. Zhang et al. [11] proved curcumin reduces proliferation and migration of MDA-MB-231 breast cancer cells by decreasing the Bcl-2/Bax ratio and inhibiting aerobic glycolysis, while enhancing apoptosis.

Although curcumin has been extensively studied in various cancer therapies, its potential to induce apoptosis in osteosarcoma cells via the mitogen-activated protein kinase (MAPK) signaling pathway by modulating glycolysis remains unexplored. Therefore, this study employed osteosarcoma cell lines U2OS and MG63 to detect changes in relevant indicators and observe apoptosis (a type of programmed cell death with distinct morphological features [12]; osteosarcoma cells also follow this pathway). Its typical hallmarks include: cell body shrinkage and cytoplasmic condensation; chromatin marginalization and condensation (pyknosis), followed by nuclear fragmentation (karyorrhexis); apoptotic bodies form via membrane budding while the plasma membrane remains intact in early stages; often accompanied by phosphatidylserine reversal. This approach elucidates the mechanism by which curcumin induces apoptosis and inhibits proliferation/migration in osteosarcoma cells, providing theoretical support and novel research directions for molecularly targeted therapies against osteosarcoma.

## 2 Materials and methods

### 2.1 Experimental materials

Osteosarcoma U2OS and MG63 cells were purchased from Sigma; U2OS and MG63-specific culture media were purchased from Sevier Bio; Curcumin was purchased from Aladdin (H136625); CCK-8 assay kit (C0038) was purchased from Biyun Tian; Annexin V-FITC staining solution was purchased from Biyun Tian (C1062S); HRP-conjugated goat anti-rabbit secondary antibody purchased from Sevier Bio (GB23303); ECL chemiluminescent reagent (G2020) purchased from Sevier Bio; Protein BCA quantification kit purchased from Biyun Tian Biotechnology (P0010); BD vertical electrophoresis apparatus and flow cytometer purchased from Shanghai Lingcheng Biotechnology Co., Ltd.; Protein-Free Rapid Blocking Solution (G2052-500 ML) and TUNEL Apoptosis Detection Kit (G1501-50T) purchased from Sevier Bio; Bax (R22708), Bcl-2 (R23309), Cleaved-Parp (R09874), HK2 (P29803), PDHA (P29803), P-JNK (P5983), P-P38 (Q16539), PKM2 (P14618), GLUT1 (P11166) antibodies were purchased from Zhengneng Bio; GAPDH monoclonal antibody (SC47724) was purchased from Santa Cruz Biotechnology, USA; purchased from ZEN bio; P38 (14064-1-AP) and JNK (66210-1-lg) were purchased from ProteinTech Group. Please refer to Supplementary Table 1 for other details.

### 2.2 Cell culture and ethical statement

U2OS and MG63 cells were grown in McCoy’s 5A medium and DMEM, respectively, with 10 % fetal bovine serum, 100 μg/mL streptomycin, 100 U/mL penicillin, at 37 °C with 5 % CO_2_. The experimental protocol was reviewed and approved by the institutional biosafety committee.

**Informed consent:** Informed consent has been obtained from all individuals included in this study.

**Ethical approval:** The research related to human use has been complied with all the relevant national regulations, institutional policies and in accordance with the tenets of the Helsinki Declaration, and has been approved by the authors’ institutional review board or equivalent committee.

### 2.3 CCK-8 assays

Prepare a “high-concentration master stock solution” using DMSO first, then perform dosing according to the “1 μL/mL method” to ensure the final DMSO concentration in all wells remains constant at ≤0.1 %. For preparing 100 mM × 1 mL: Weigh 36.84 mg of powder and dissolve in 1 mL DMSO (store in light-protected, −20 °C aliquots). For 10 mL preparations: 200 µM = 20 µL stock + 80 µL additional DMSO 400 µM = 40 + 60 600 µM = 60 + 40 800 µM = 80 + 20 Top up to 10 mL with respective medium. Dosing: Add 90 µL medium + 10 µL corresponding working solution per well → final concentrations 20/40/60/80 µM, final DMSO = 0.10 %. Vortex during addition to prevent localized crystallization. Perform operations in the dark; dissolve at room temperature. Seed U2OS and MG63 cells into 96-well plates (2 × 10^3^ cells/well) in culture medium and incubate for 24 h. Cells were divided into five groups: control and curcumin-treated groups (20, 40, 60, and 80 μmol/L), with five replicates per group. After 24 h of treatment, add 10 µL of CCK-8 reagent to each well. After an additional 2 h of incubation, absorbance was measured at 450 nm using a microplate reader (Bio-Rad Laboratories, USA). Cell viability was calculated using the following formula:

$$\text{Cell viability }\left(\%\right)=\left(\frac{\text{Absorbance}\;\text{of}\;\text{treatment}\;\text{group}}{\text{Absorbance}\;\text{of}\;\text{the}\;\text{control}\;\text{group}}\;\right)\times 100\;\%$$

### 2.4 Western blot (WB) detection

MG63 and U2OS cells (1 × 10^6^ cells/mL) were seeded into 25 cm^2^ culture flasks. After attachment, cells were placed in untreated controls, or treated with 20 or 40 μmol/L curcumin for 24 h. Following treatment, cells were placed on ice and lysed with PMSF-containing protein lysis buffer for 2 h, then centrifuged at 12,000 rpm for 15 min. Collect the supernatant and determine protein content using a BCA assay kit. Denature proteins in an equal volume of 5 × loading buffer at 100 °C for 10 min, then separate by 10 % SDS-PAGE and transfer to a PVDF membrane. Incubate membranes in rapid blocking buffer for 2 h, then incubate with primary antibodies against pro-apoptotic protein (Bax), anti-apoptotic protein (Bcl-2), apoptotic marker (cleaved-PARP), hexokinase 2 (HK2), glucose transporter 1 (GLUT1), pyruvate kinase M2 (PKM2), pyruvate dehydrogenase E1α subunit (PDHA), and glyceraldehyde-3-phosphate dehydrogenase (GAPDH) overnight at 4 °C. After washing with phosphate-buffered saline (PBS), membranes were incubated with secondary antibodies at room temperature for 2 h, visualized using an enhanced chemiluminescence (ECL) kit, and quantified with ImageJ software. Each experiment was replicated three times. GAPDH served as the loading control.

### 2.5 The TUNEL assay detects DNA fragmentation in cells following treatment with curcumin at various concentrations

Seed U2OS and MG63 cells at a density of approximately 5 × 10^4^ cells/well onto coverslips in 24-well plates. After reaching 70–80 % confluence, replace the medium with serum-free medium. Treat cells with 0, 20, or 40 μmol/L curcumin for 24 h, then wash twice with PBS. Fix cells at room temperature with 4 % paraformaldehyde for 20 min, then wash three times with PBS for 5 min each. Permeabilize with 0.1 % Triton X-100 for 10 min, followed by another PBS wash. Apply the TUNEL reaction mixture at 37 °C in the dark for 1 h. After washing, counterstain nuclei with 1 μg/mL DAPI for 5 min in the dark. Rinse cells again and mount with a coverslip in a mounting medium. Image fluorescence signals under a fluorescence microscope in the FITC channel (TUNEL-positive cells appear green fluorescence) and the DAPI channel (blue nuclei).

### 2.6 Cell scratch assay

Seed OS cells (U2OS, MG63) uniformly into 6-well plates, ensuring even cell distribution and sufficient cell numbers per well to allow complete monolayer formation within 24 h. Culture cells until a 100 % confluent monolayer is established. Using a sterile 200 μL pipette tip, create straight, uniform scratches across the cell monolayer. To enhance experimental reproducibility, create three parallel scratches in each well. After scratching, gently wash the plate three times with PBS to remove suspended cell debris and detached cells, ensuring a clear observation area. Subsequently, incubate cells with curcumin at concentrations of 0, 20, or 40 μmol/L in serum-free medium. Image the scratch area at 0 and 24 h post-treatment to assess scratch closure rate. The purpose of this assay is to evaluate whether curcumin influences OS cell migration by altering cellular metabolic status. Measurements were performed using ImageJ software with an inverted microscope (manufacturer: UOP, model number: DSY5000X).

### 2.7 Detection of apoptosis in MG63 and U2OS cells by flow cytometry (FCM)

U2OS and MG63 cells in logarithmic growth phase were adjusted to a concentration of 5 × 10^4^ cells/mL and seeded into 6-well plates. Curcumin at concentrations of 0, 20, and 40 μmol/L was added to each group, followed by continued culture for 24 h. Subsequently, cells were harvested, washed with PBS, and centrifuged for 5 min to pellet. The pellet was resuspended in 200 μL binding buffer containing 10 μL Annexin V-FITC and 5 μL PI. After mixing, the mixture reacted for 30 min at room temperature in the dark. Finally, the levels of programmed cell death in each group were detected by flow cytometry. After the incubation period, the stained cells were resuspended in HEPES buffer and analyzed using a FACS Calibur 440E flow cytometer (Shanghai Lingcheng Biotechnology Co., Ltd.) according to standard procedures [13]. A total of 10,000 gated events were analyzed using Cell Quest software.

### 2.8 Network pharmacology and pathway enrichment analysis

To further elucidate the potential molecular mechanism of curcumin’s action on osteosarcoma, a systematic analysis was conducted using network pharmacology and molecular docking techniques. Curcumin targets were predicted using the PubChem, SwissTargetPrediction, TargetNET, and CHEMBL databases. Osteosarcoma-related targets were screened in the GeneCards, OMIM, TTD, DrugBank, and PharmGKB databases using “Osteosarcoma” as a keyword; the intersection of these databases yielded 92 common targets. A protein-protein interaction network (PPI) was constructed using the STRING database, and topology analysis was performed using Cytoscape 3.9.1 to identify core nodes such as MAPK14 (p38 MAPK), AKT1, TP53, STAT3, and BCL2. Further GO and KEGG enrichment analyses were performed using the David database, and molecular docking was performed using AutoDock Vina. The molecular docking results visualized the potential affinity between curcumin and key proteins in the MAPK pathway.

### 2.9 Measurement of glucose consumption, lactate production, and intracellular ATP levels

U2OS and MG63 cells were seeded in 6-well plates and treated with curcumin at the indicated concentrations for 24 h. After treatment, culture supernatants were collected and centrifuged at 12,000 × *g* for 10 min at 4 °C to remove cell debris. Cell numbers in each well were counted for normalization.

Glucose concentration in the supernatant was determined using a glucose oxidase–peroxidase colorimetric assay kit (Ruixin Biotechnology Co., Ltd., Quanzhou, China; Cat. No. RXWB0164-96) according to the manufacturer’s protocol. Absorbance was measured using a microplate reader (Model, Manufacturer) at 505 nm (or the wavelength specified by the kit). Glucose consumption was calculated as: glucose consumption rate = ([Glc]0 − [Glc]*t*) × *V*/(*N* × *t*), where [Glc]0 and [Glc]*t* represent glucose concentrations at 0 h and time *t*, respectively, *V* is the medium volume, *N* is the cell number, and t is the incubation time.

Lactate concentration in the supernatant was measured using a lactate oxidase (LA) colorimetric assay kit (Wuhan Yilairuit Biotechnology Co., Ltd.; Cat. No. E-BC-K044-M). Briefly, 50 μL of sample or standard was added to each well, followed by 100 μL of working reagent, and incubated at 37 °C in the dark for 15–30 min. Absorbance was read at 530 nm (or as specified by the kit), and lactate concentration was calculated using a standard curve. Lactate production was calculated as: lactate production rate = ([Lac]*t* − [Lac]0) × *V*/(*N* × *t*).

Intracellular ATP levels were quantified using a luciferase-based ATP assay kit (Ruixin Biotechnology Co., Ltd., Quanzhou, China; Cat. No. RX2D235796). Cells were washed with cold PBS and lysed using the provided lysis buffer. After centrifugation (12,000 × *g*, 5 min, 4 °C), the supernatant was mixed with ATP detection reagent, and luminescence was measured immediately using a or microplate reader (Bio-Rad Microplate Reader (USA)). ATP values were normalized to total protein quantified by a BCA protein assay (Biyuntian Biotechnology Co., Ltd.; Cat. No. P0009) and expressed as relative ATP content.

### 2.10 Statistical analysis

Data analysis was performed using GraphPad Prism 9.0 software (GraphPad Software, San Diego, USA). All experiments were independently repeated at least three times (*n* ≥ 3). Experimental data are expressed as mean ± standard deviation (Mean ± SD). Independent samples *t*-tests were used for comparisons between two groups; one-way ANOVA was used for comparisons of three or more groups, and appropriate post-hoc tests (LSD or Tukey test) were selected based on the homogeneity of variance results. A *P*-value < 0.05 was considered statistically significant, and significance levels were expressed as *P < 0.05, **P < 0.01, and ***P < 0.001.

## 3 Results

### 3.1 Network pharmacology and molecular docking results

**Figure 1:**
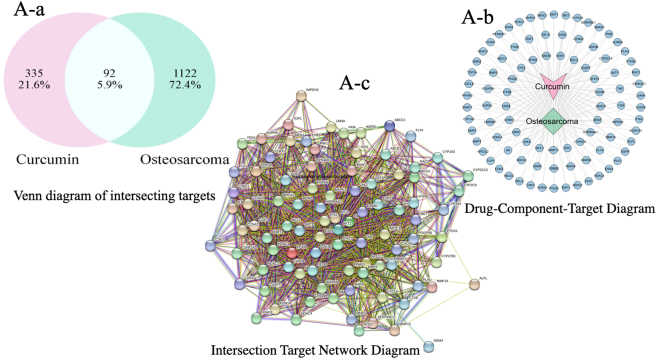
Core target network analyzed via Cytoscape topology metrics. (A-a) Core target network based on cytoscape analysis. Node size is proportional to degree value, while color intensity represents betweenness centrality. MAPK14 (P38 MAPK) occupies the network center, forming a highly connected subnetwork with BCL2, TP53, STAT3, and others, indicating P38 MAPK’s pivotal role in the curcumin action network. (A-b) Curcumin forms a dense network with osteosarcoma through numerous shared targets, suggesting curcumin exhibits a typical “multi-target” regulatory profile against osteosarcoma; Among these, molecules such as TP53, TNF, AKT1, EGFR, STAT3, and MMPs – closely associated with cell proliferation, apoptosis, invasion/metastasis, and the tumor microenvironment – occupy central positions in the network. These may represent key pivotal targets through which curcumin exerts its anti-osteosarcoma effects. (A-c) The interactions among nodes within this core network are exceptionally tight, exhibiting high connectivity and strong bridging effects. This indicates that these genes occupy pivotal positions in information transmission and signal integration within the overall network, potentially constituting a key regulatory module for curcumin intervention in osteosarcoma. Genes such as TP53, TNF, AKT1, EGFR, STAT3, members of the MAPK family, and various MMPs are closely associated with cell proliferation, apoptosis, inflammatory responses, and tumor invasion/metastasis. Their “high centrality” within the network provides a key direction for subsequent mechanism validation experiments targeting these core pathways.

**Figure 2:**
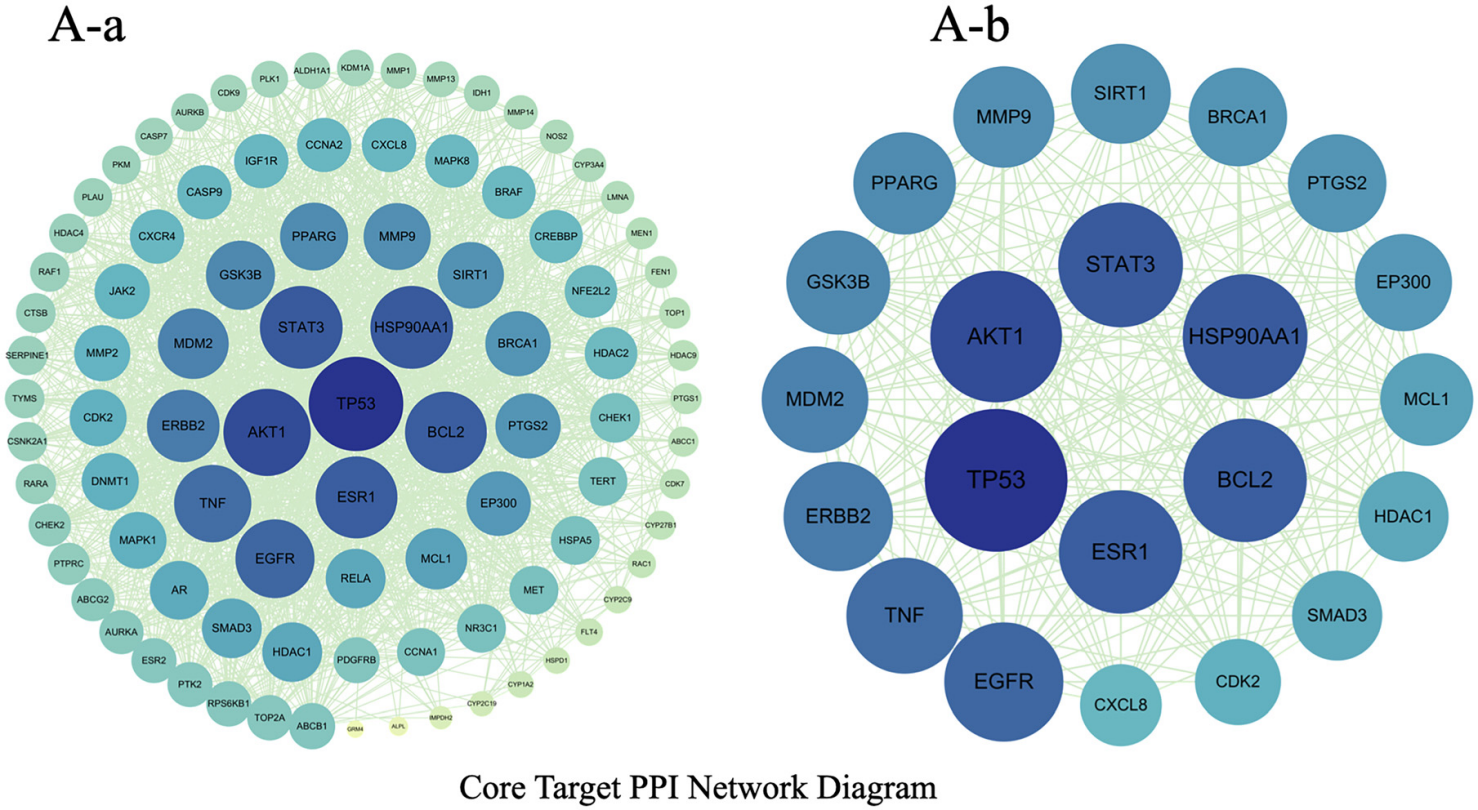
Protein-protein interaction (PPI) network constructed from the STRING database. Nodes represent proteins, and edges indicate interaction relationships. Genes such as P38 MAPK (MAPK14), AKT1, TP53, STAT3, and BCL2 exhibit high connectivity within the network, indicating potential core hubs. (A-a) Curcumin forms a dense network with osteosarcoma through numerous common targets, indicating its characteristic “multi-target” regulatory profile against osteosarcoma. Key molecules such as TP53, TNF, AKT1, EGFR, STAT3, and MMPs – closely associated with cell proliferation, apoptosis, invasion/metastasis, and the tumor microenvironment – occupy central positions within this network. These molecules may constitute key pivotal targets through which curcumin exerts its anti-osteosarcoma effects. (A-b) Within this subnetwork, the number of nodes is significantly lower than in the original network, yet the number of edges remains at a high level. This indicates substantial direct or indirect connections among the retained 22 genes, exhibiting distinct characteristics of “high clustering + high connectivity.” These nodes exhibit higher degree values, shorter average path lengths, and greater betweenness centrality in the original network. This indicates they serve as both highly connected nodes within local modules and as information “bridges” between different functional modules, occupying a central position in network structure and signal transmission.

**Figure 3:**
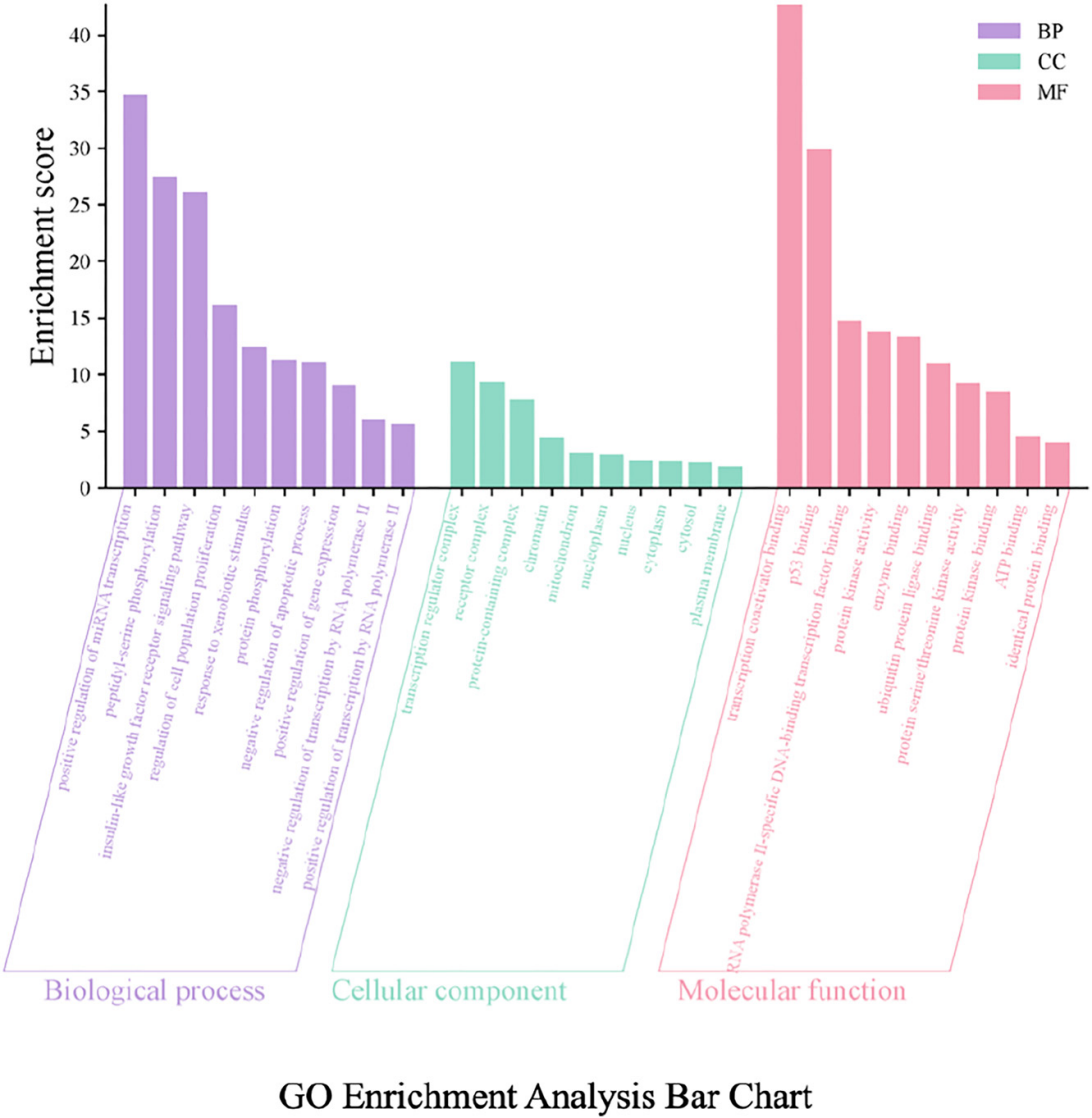
GO enrichment analysis plot. In terms of BP, significantly enriched entries primarily include protein phosphorylation, peptidyl-serine phosphorylation, regulation of cell population proliferation, negative regulation of apoptotic process, positive regulation of gene expression, and response to xenobiotic stimulus. This suggests these targets are extensively involved in protein phosphorylation cascades, regulation of cell population proliferation, apoptosis suppression, and responses to exogenous compounds/stress (e.g., drugs). This aligns strongly with the biological characteristics of osteosarcoma, including high proliferation, anti-apoptotic properties, and altered drug sensitivity. For CC, target genes were predominantly enriched in the nucleus, nucleoplasm, chromatin, transcription regulator complex, protein-containing complex, mitochondria, and plasma membrane. This indicates functions involving both nuclear chromatin and transcription regulatory complexes, as well as cytoplasmic/mitochondrial and membrane receptor-associated structures, reflecting a complete spatial framework from membrane receptor signal sensing and cytoplasmic cascade transduction to nuclear transcription regulation. MF analysis revealed significant enrichment in protein kinase activity, protein serine/threonine kinase activity, protein kinase binding, and ATP binding. Concurrently, entries such as p53 binding, RNA polymerase II-specific DNA-binding transcription factor binding, transcription coactivator binding, and ubiquitin protein ligase binding were present, suggesting these targets play crucial roles in kinase activity regulation, energy utilization, p53-mediated stress/apoptotic pathways, and ubiquitin-mediated degradation and transcriptional regulation. Apoptosis pathways, ubiquitin-mediated degradation, and transcriptional regulation. Collectively, the GO analysis results support the concentrated distribution of intersecting target genes along a functional continuum encompassing “membrane receptor–protein phosphorylation cascade–nuclear transcription regulation–cell proliferation and apoptosis.” This provides functional evidence for curcumin’s multi-target regulation of osteosarcoma cell fate and drug responsiveness.

**Figure 4:**
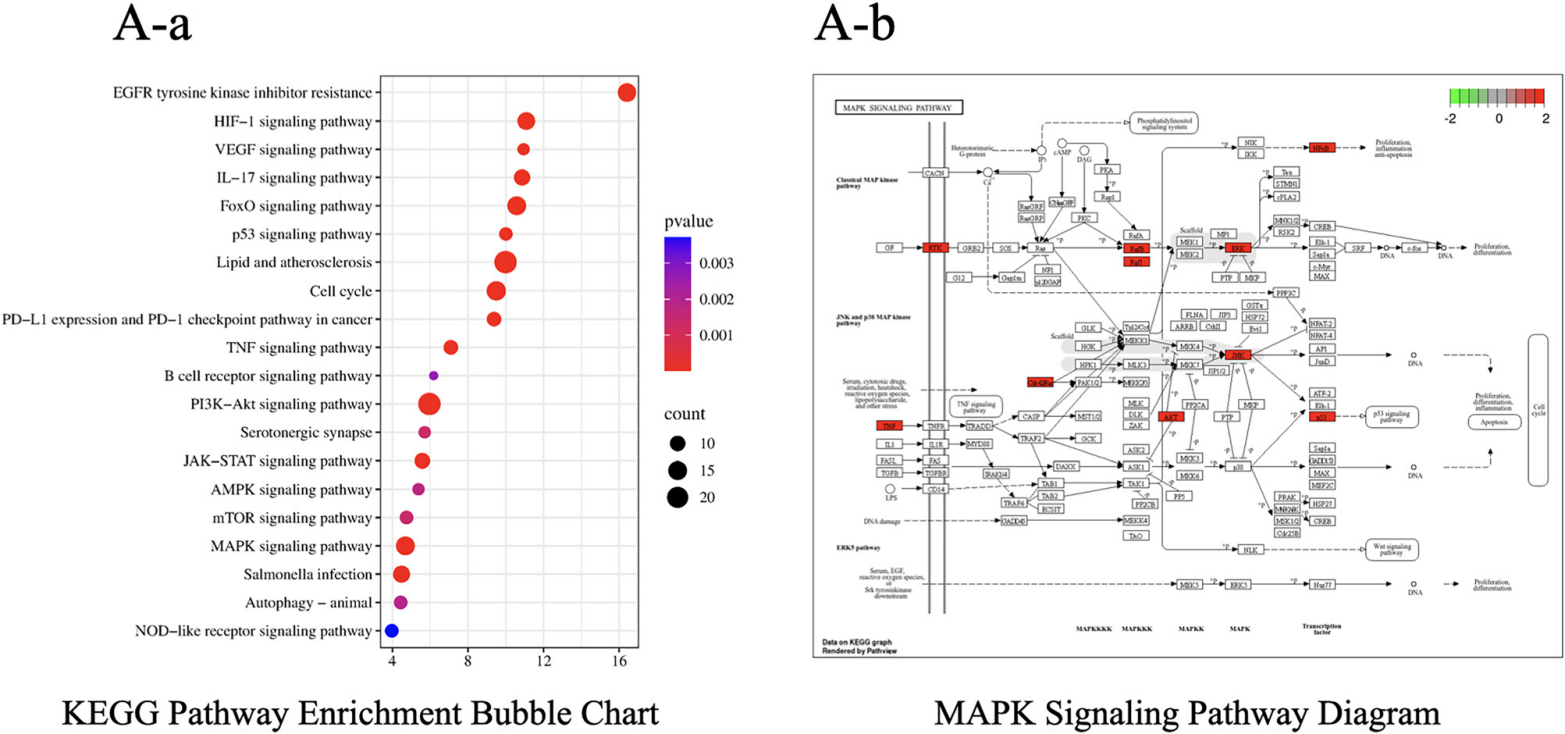
KEGG MAPK signaling pathway enrichment plot. (A-a) The top 20 pathways are highly concentrated in core processes such as tumor-related signal transduction, cell proliferation and apoptosis regulation, metabolism, and inflammation/immunity. These include EGFR tyrosine kinase inhibitor resistance, HIF-1, VEGF, IL-17, TNF, JAK-STAT, PI3K-Akt, MAPK, mTOR, AMPK, FoxO, p53, as well as cell cycle, PD-1/PD-L1 immune checkpoint pathways, B-cell receptor signaling, autophagy-autophagy, NOD-like receptor signaling, lipid and atherosclerosis, serotonergic synapse, and Salmonella infection pathways. Pathways with higher fold enrichment and larger bubbles (e.g., PI3K-Akt, MAPK, TNF, HIF-1, p53, Cell Cycle, Autophagy, etc.) indicate a higher concentration of overlapping genes within these pathways and a significantly increased enrichment factor. This suggests that curcumin’s targets in osteosarcoma primarily cluster within key signaling networks regulating cell proliferation/apoptosis, autophagy, energy metabolism reprogramming, inflammation, and the immune microenvironment. (A-b) Mapping results reveal that the intersecting target genes are widely distributed across the MAPK signaling pathway, covering the classical MAPK cascade, JNK/p38 branches, and key nodes interacting with the p53 pathway (Figure X). Upstream, molecules such as TNF and other cytokines are highlighted, suggesting that inflammation and stress stimuli are important triggers for activating this pathway. Midstream, multi-tiered kinase cascades (e.g., MAPKKK–MAPKK–MAPK) and stress-responsive MAPK members like JNK and p38 were marked, indicating that intersecting targets primarily participate in amplifying and transducing stress signals. Downstream, transcription factor nodes such as TP53 were also enriched, connecting to effector pathways including cell cycle arrest, apoptosis, and differentiation.

Curcumin shares 427 potential targets with osteosarcoma-related targets, which number 1,214, yielding 92 overlapping targets (Figure 1). A protein-protein interaction (PPI) network was constructed for these overlapping targets using the STRING database (Figure 2), with MAPK14 (P38 MAPK) exhibiting the highest centrality. GO enrichment analysis yielded 592 annotated terms, primarily involving transcriptional regulation, protein phosphorylation, and apoptosis (Figure 3). KEGG enrichment identified 145 pathways, with the MAPK signaling pathway showing the highest enrichment (Figure 4).

**Figure 5:**
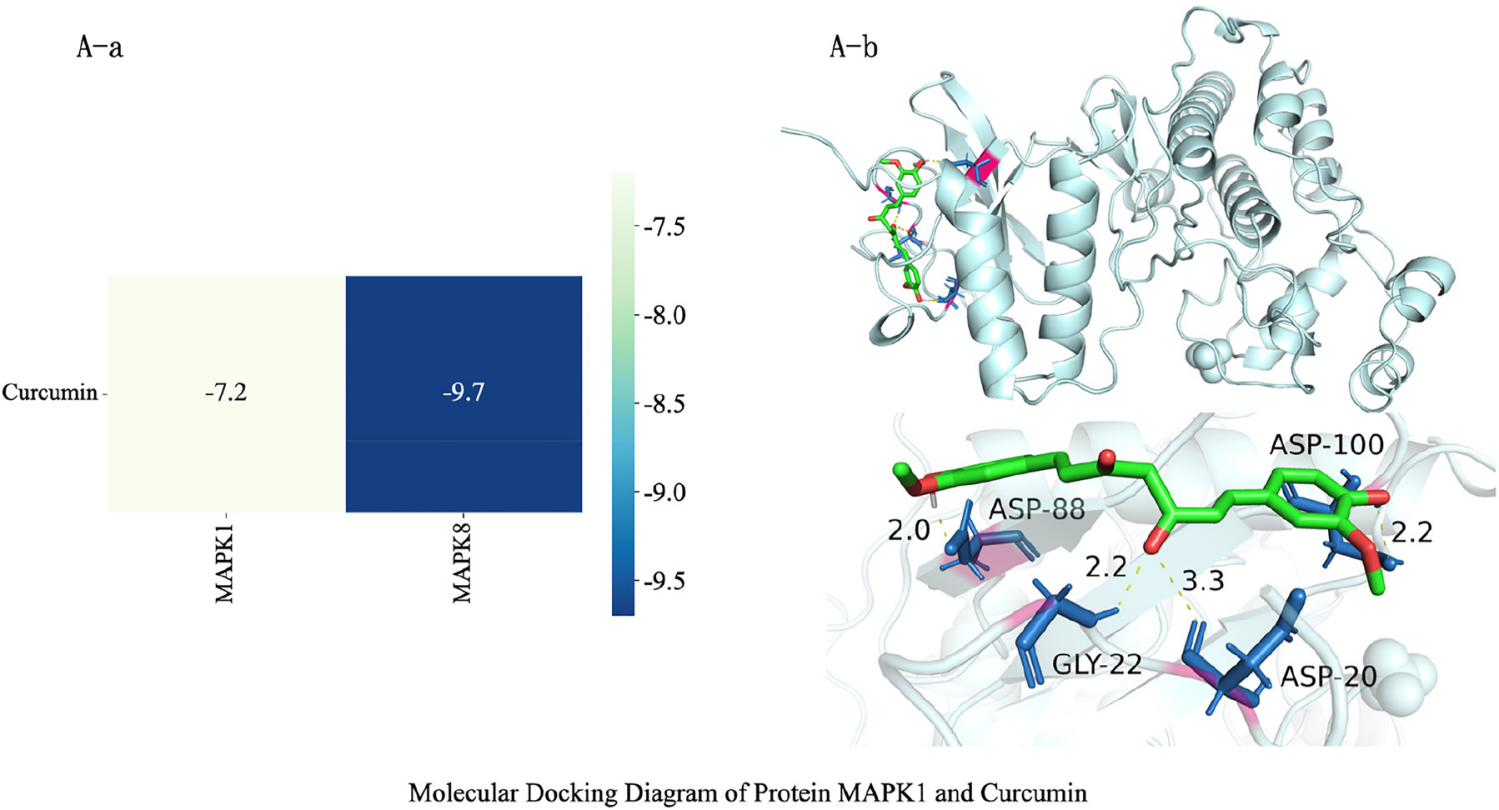
Molecular docking diagram of curcumin and MAPK1 protein. (A-a)Molecular docking results indicate that curcumin exhibits strong binding affinity with both MAPK1 and MAPK8, with docking scores of −7.2 and −9.7 kcal/mol, respectively. Generally, docking energies below approximately −7.0 kcal/mol indicate strong potential binding capacity. Thus, curcumin exhibits favorable interactions with both proteins, with significantly higher affinity for MAPK8 than for MAPK1. In the heatmap, darker colors indicate lower binding energy and higher affinity. The curcumin–MAPK8 combination appears in a darker shade, further confirming it as a more likely preferred target. At the molecular level, this suggests curcumin may directly bind and regulate MAPK/JNK-related kinase activity, thereby influencing downstream MAPK signaling pathways and their mediated processes of cell proliferation, apoptosis, and stress responses. It is important to emphasize that molecular docking results reflect only theoretical binding trends; further validation of actual biological effects through *in vitro* kinase activity assays and binding kinetics experiments is required. (A-b) The figure shows curcumin (green rod-shaped) stably embedded within the catalytic pocket of MAPK1. Surrounding helices and folded sheets form a relatively enclosed groove structure, “enveloping” the small molecule near the enzyme’s active site. This provides the spatial foundation for forming multi-point interactions, consistent with the previously obtained docking energy (−7.2 kcal/mol). This indicates that this binding conformation possesses high stability. The structural hydrogen-bond network and pocket adaptability effectively explain curcumin’s favorable affinity in docking scores, supporting its potential to regulate the MAPK signaling pathway through direct binding to MAPK1. However, this inference requires further validation through subsequent kinase activity assays and cellular-level functional experiments.

**Figure 6:**
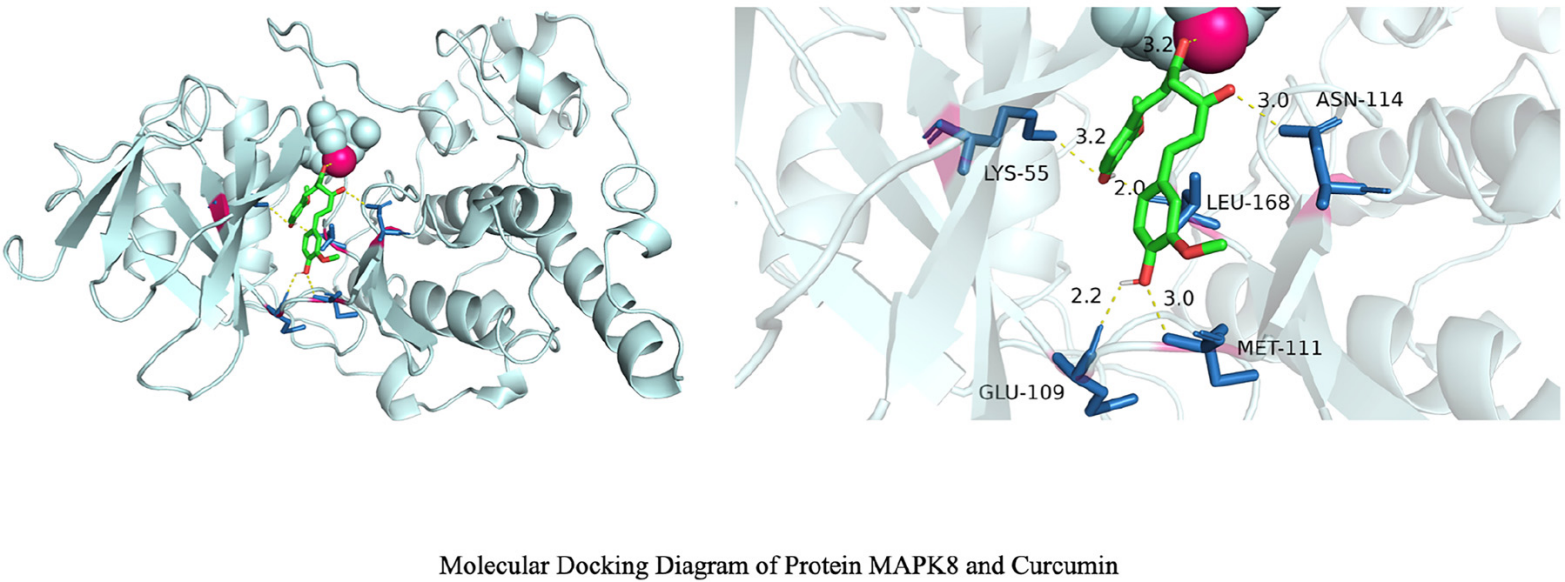
Molecular docking diagram of curcumin and MAPK1 protein. The figure shows curcumin (green rod) stably embedded within the catalytic pocket of MAPK8 (JNK1), extending along a narrow channel formed by multiple *β*-folds and helices. Both ends of the ligand approach the pocket entrance, while its central segment resides in the core region. The surrounding protein secondary structures form a relatively enclosed microenvironment, which helps maintain the ligand conformation and multi-point interactions. This aligns with its docking score of −9.7 kcal/mol, suggesting MAPK8 exhibits stronger affinity for curcumin. Compared to MAPK1, curcumin forms more hydrogen bonds with MAPK8, exhibits a more concentrated spatial distribution, and demonstrates lower docking energy, indicating that MAPK8 shows stronger structural complementarity and binding affinity for curcumin. Combined with prior network pharmacology and pathway enrichment results, MAPK8 (JNK pathway) itself occupies a pivotal position in stress and apoptosis regulation within osteosarcoma-related MAPK signaling. This molecularly supports the hypothesis that “curcumin may directly target MAPK8/JNK to regulate downstream apoptosis and stress-related signaling.” However, this conclusion requires further validation through subsequent kinase activity assays and cellular functional experiments.

Molecular docking revealed binding energies of −6.3 kcal/mol and −5.8 kcal/mol for curcumin with MAPK1 and MAPK8 proteins, respectively (Figures 5 and 6), indicating stable binding of curcumin to key MAPK pathway proteins. Comprehensive results suggest that curcumin may regulate glycolysis and apoptosis through the p38 MAPK signaling branch, consistent with the mechanisms observed in cellular experiments.

### 3.2 CCK8 detection of the effect of curcumin on the survival rate of osteosarcoma cells

**Figure 7:**
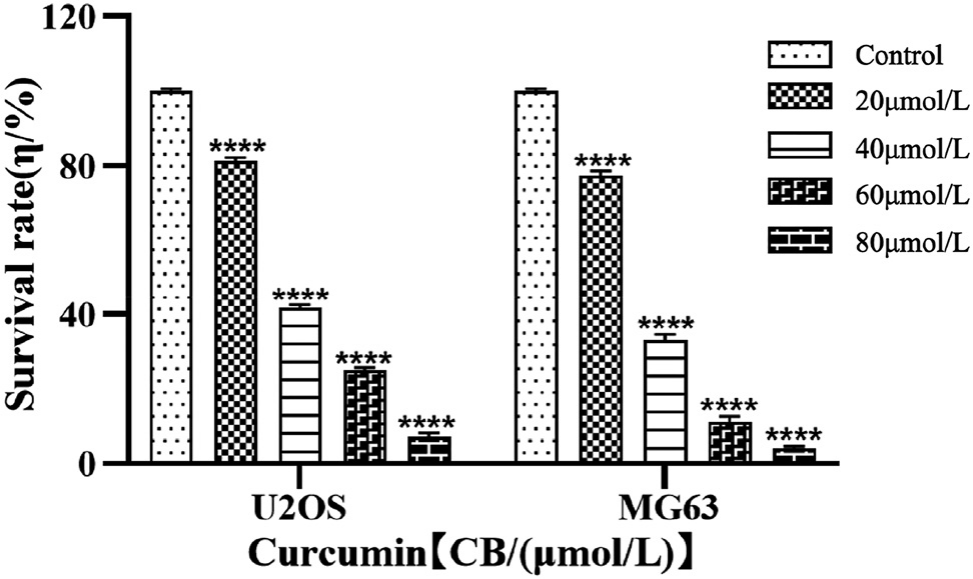
Effects of different curcumin concentrations on the survival rates of U2OS and MG63 cells. ***P < 0.001.

After treating U2OS and MG63 cells with curcumin at different concentrations for 24 h, the CCK-8 assay results in Figure 7 showed that cell viability decreased with increasing drug concentration in a dose-dependent manner compared to the control group (*P* < 0.05, *P* < 0.01). The IC50 values were 32.6 μmol/L for MG63 cells and while the U2OS IC_50_ was 37.3 μmol/L.

### 3.3 Flow cytometry (FCM) analysis of curcumin’s effect on apoptosis in osteosarcoma cells

**Figure 8:**
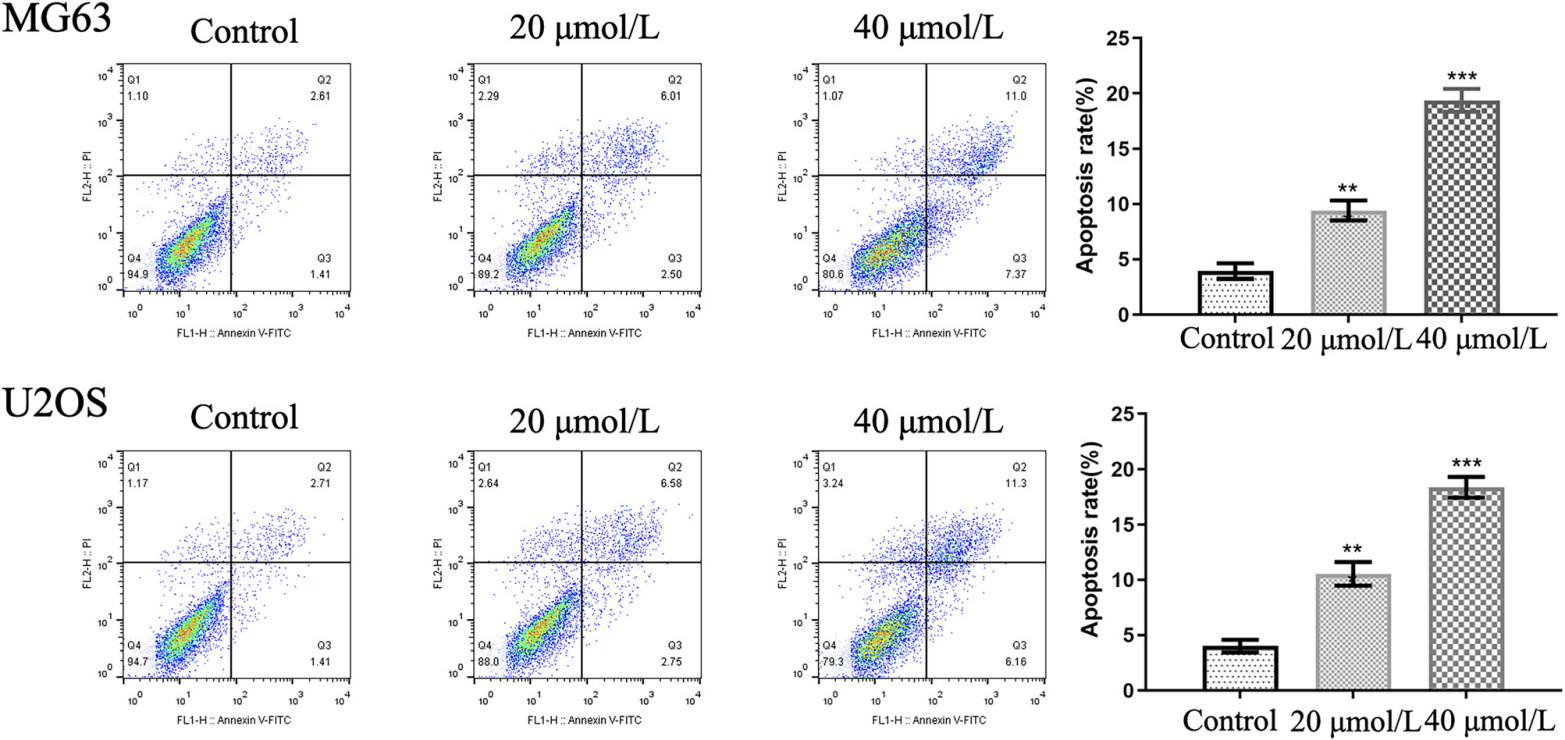
Effects of different concentrations of curcumin on apoptosis in U2OS and MG63 cells. **P < 0.01, ***P < 0.001.

Apoptosis in U2OS and MG63 cells was assessed using Annexin V–FITC/PI double staining. With increasing curcumin concentrations, the proportion of Annexin V^+^/PI^+^ cells significantly increased, indicating a dose-dependent rise in programmed cell death (Figure 8).

### 3.4 Effects of curcumin on glycolysis in osteosarcoma cells

**Figure 9:**
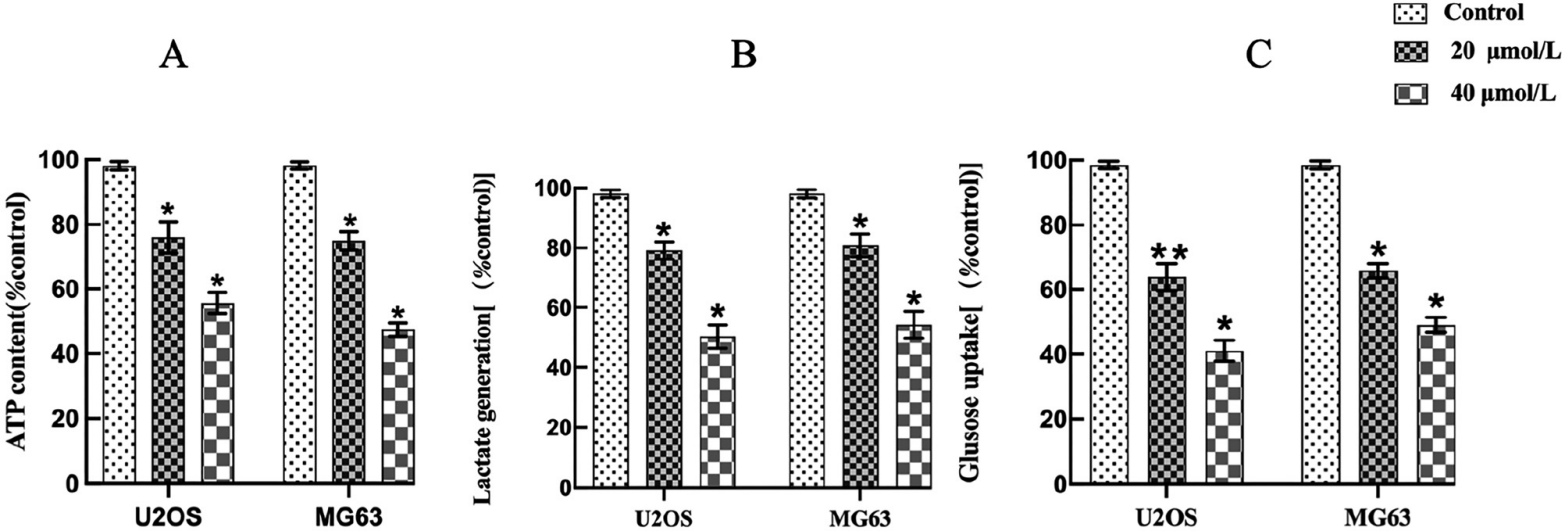
Effects of different curcumin concentrations on the glycolytic pathway in U2OS and MG63 cells (*x* ± *s*, *n* = 3) (A): ATP; (B): GLU; (C): LAC. *P < 0.05, **P < 0.01.

Glycolytic activity in U2OS and MG63 cells incubated with curcumin was assessed by measuring intracellular adenosine triphosphate (ATP) content, glucose uptake, and lactate production. ATP levels were determined to evaluate intracellular energy status; glucose uptake was measured using the glucose oxidase method to assess utilization of the cellular carbohydrate substrate; lactate production was measured via the lactate dehydrogenase assay to detect changes in glycolytic end products. Curcumin significantly reduced ATP production, glucose uptake, and lactate levels in a dose-dependent manner (*P < 0.05; Figure 9). These findings indicate that curcumin inhibits glycolysis, thereby limiting cellular energy production and impairing cell proliferation and survival.

### 3.5 Effects of curcumin on the migration capacity of U2OS and MG63 cells

**Figure 10:**
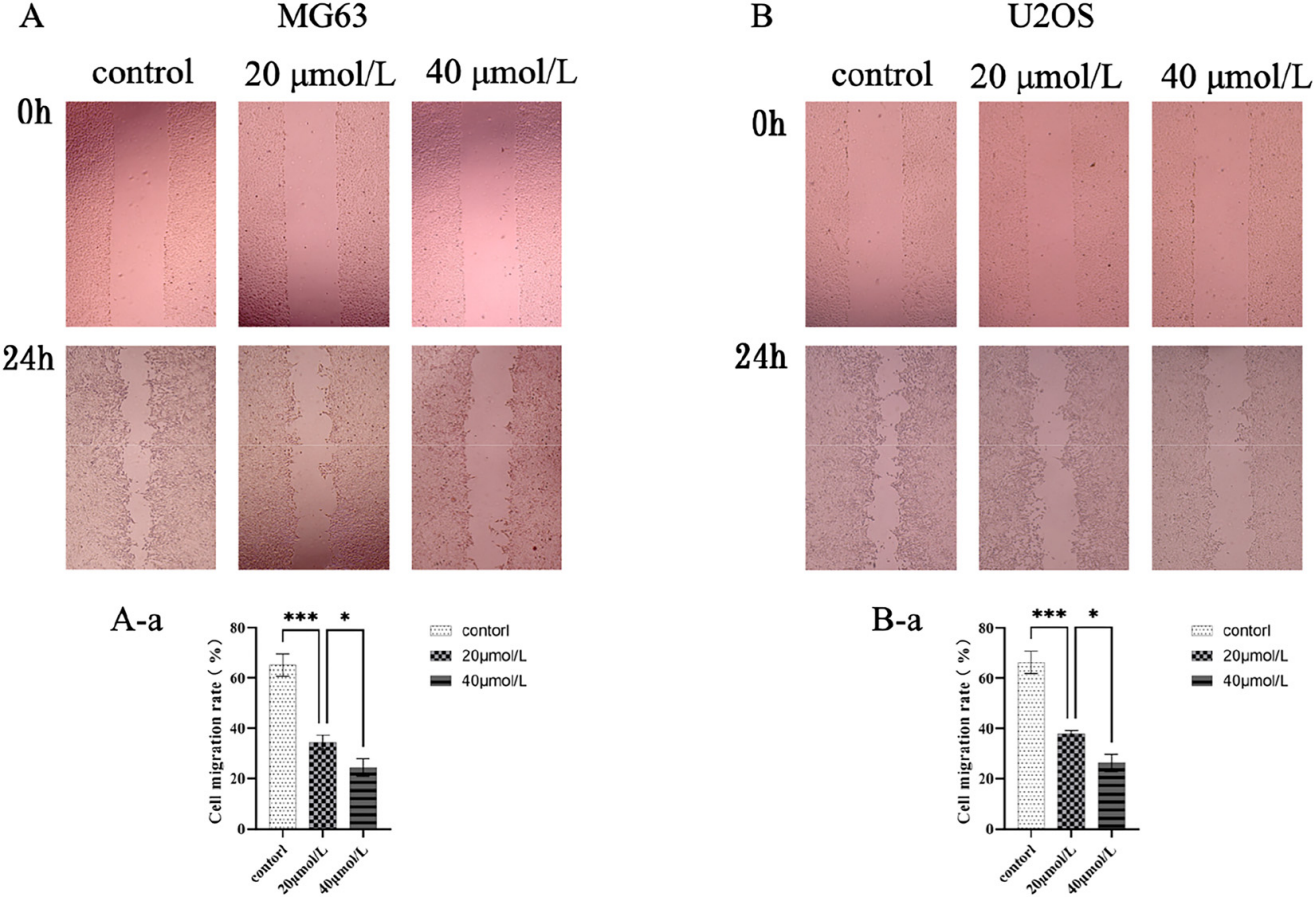
Effects of curcumin on migration capacity and morphology of U2OS and MG63 cells (50 × magnification) and quantification of MAPK phosphorylation levels after curcumin treatment. (A) Effects of different curcumin concentrations (0, 20, 40 μmol/L) on MG63 cell migration after 24 h. (B) Effects of different curcumin concentrations (0, 20, 40 μmol/L) on U2OS cell migration after 24 h. A-b (MG63): Quantitative analysis of the scratch assay. B-a (U2OS): Quantitative analysis of the scratch assay. *P < 0.05, ***P < 0.001 versus control group.

Cultured U2OS and MG63 cells in curcumin-containing medium for 24 h. Assessed cell migration via scratch assay, imaging at 0 and 24 h using an inverted microscope. Observed cell movement toward the scratch site and migration-related morphological changes (e.g., pseudopod formation and cell polarity) to evaluate curcumin’s impact on migratory behavior. Control cells exhibited normal morphology, good adhesion, and minimal migration inhibition. Treatment with 20 μmol/L curcumin reduced cell migration, showing early signs of delayed scratch healing and morphological alterations. At 40 μmol/L curcumin, cell migration was significantly reduced or even completely inhibited. Cells treated with both 20 μmol/L and 40 μmol/L curcumin exhibited reduced cell numbers, along with pronounced shrinkage, detachment, and floating, suggesting apoptosis (Figure 10). These findings indicate that curcumin inhibits OS cell migration in a dose-dependent manner, potentially achieved by inducing apoptosis and disrupting cellular structural dynamics.

### 3.6 Western blot analysis of curcumin’s effects on glycolysis and apoptosis-related protein expression in U2OS and MG63 cells

**Figure 11:**
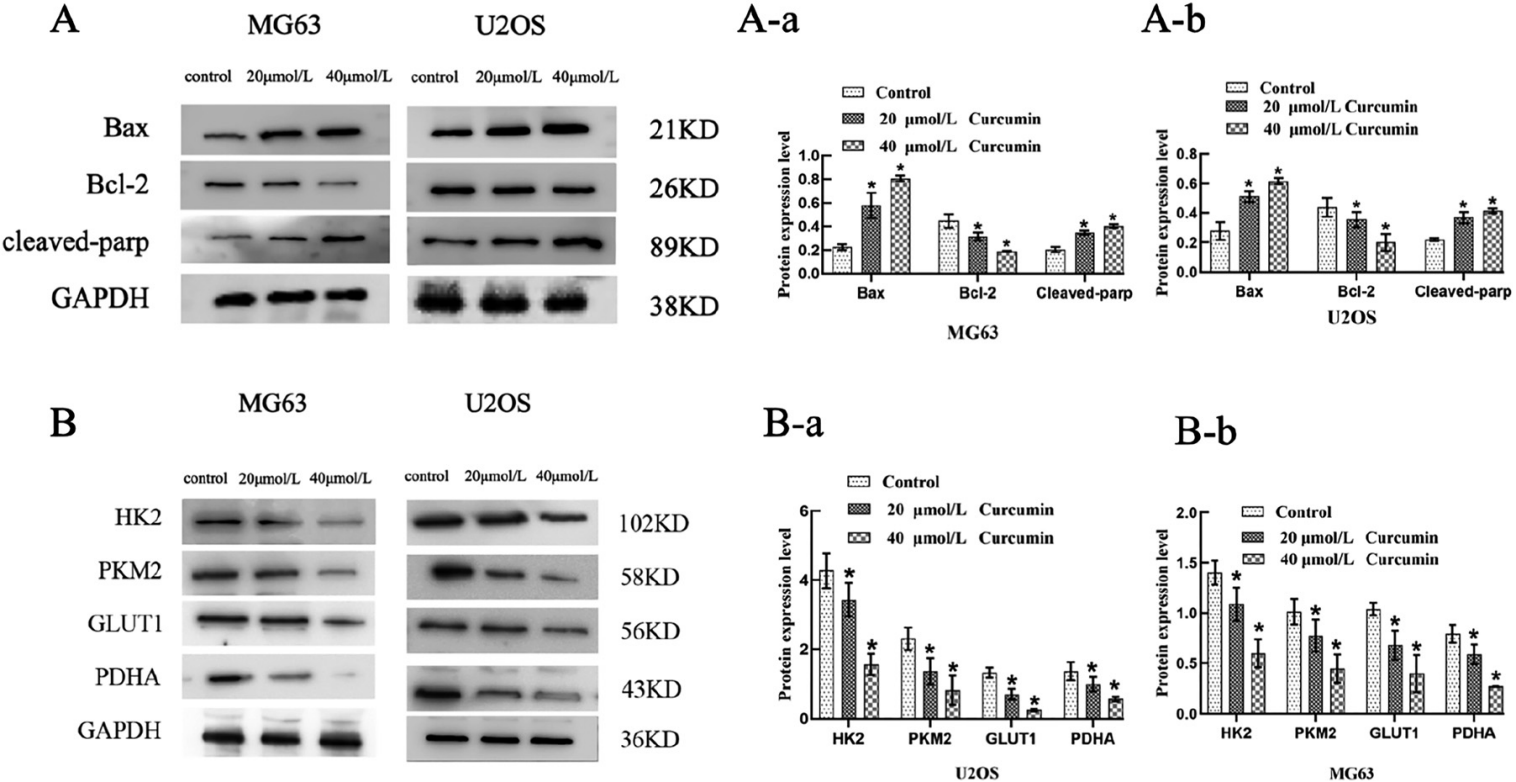
Effects of different concentrations of Cur on glycolysis and apoptosis-related proteins in U2OS and MG63 cell. (A) Effects of different curcumin concentrations on glycolysis and apoptosis-related proteins in U2OS and MG63 cells (*x* ± *s*, *n* = 3). *P < 0.05 versus control group. (A-a) MG63 cells: Expression levels of Bax, Bcl-2, and cleaved-PARP proteins. (A-b) MG63 cells: Bax, Bcl-2, and cleaved-PARP protein expression levels. (B) Effects of curcumin on apoptosis-related protein levels in MG63 and U2OS cells (*x* ± *s*, *n* = 3). *P < 0.05 versus control group. (B-a) U2OS cells: HK2, PKM2, GLUT1, PDHA protein expression levels. (B-b) MG63 cells: HK2, PKM2, GLUT1, PDHA protein expression levels.

WB analysis revealed that curcumin downregulated the expression of glycolysis-related proteins HK2 and PKM2 in both MG63 and U2OS cells in a dose-dependent manner, while simultaneously upregulating the expression of pro-apoptotic protein Bax and cleaved PARP (Figure 11, **P < 0.01). These findings indicate that curcumin inhibits glycolytic metabolism in OS cells and promotes their apoptosis.

### 3.7 Western blot analysis of curcumin’s effects on the expression of proteins related to the JNK/P38 signaling pathway in U2OS and MG63 cells

**Figure 12:**
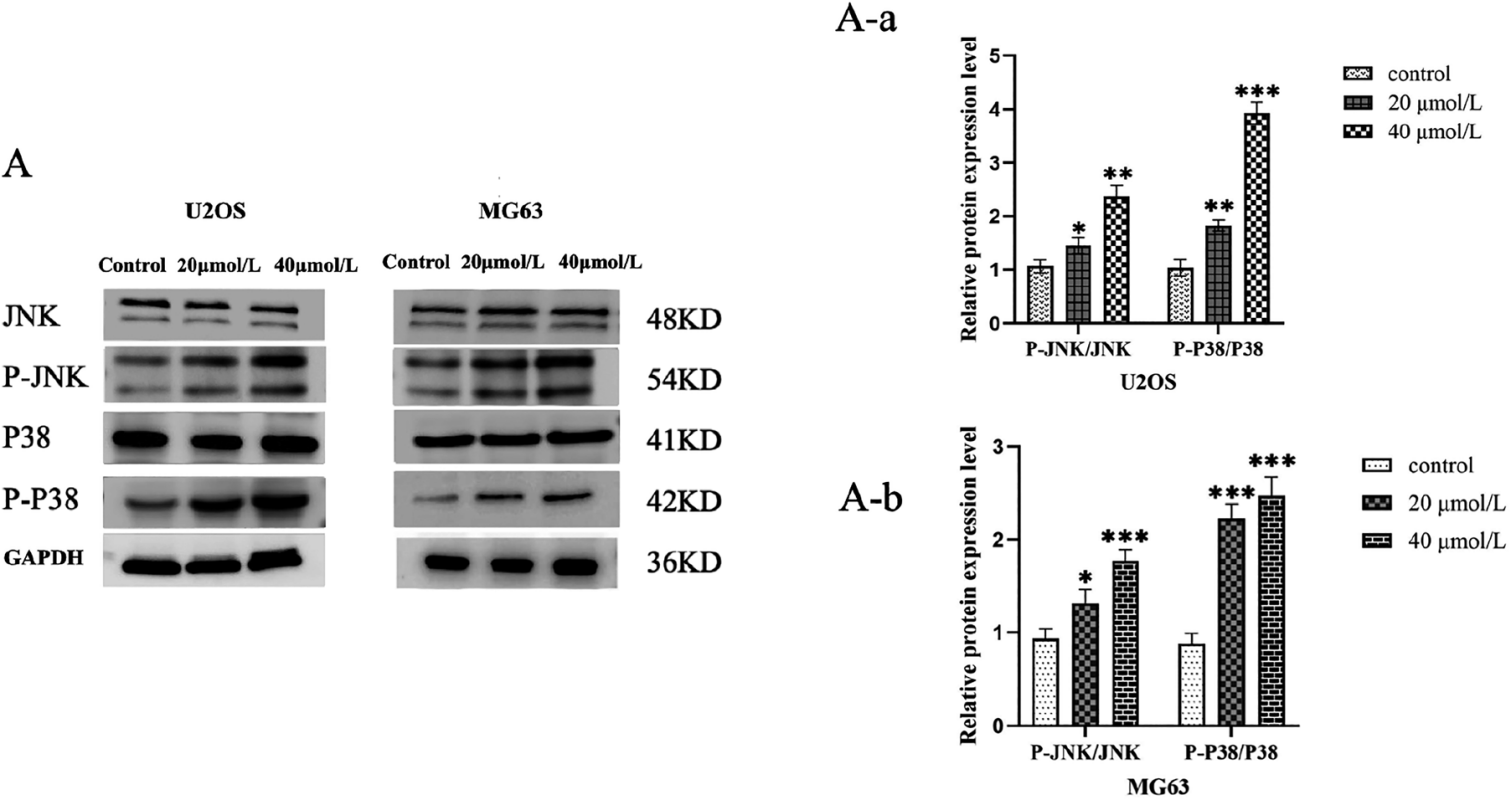
Effects of different curcumin concentrations on JNK/P38 signaling pathway protein expression in U2OS and MG63 cells. Curcumin activates the JNK/p38 MAPK signaling pathway in osteosarcoma cells. Representative western blots and quantitative densitometry of phosphorylated JNK (p-JNK), total JNK, phosphorylated p38 (p-p38), and total p38 in U2OS and MG63 cells treated with curcumin (0, 20, and 40 μM) for 24 h. Total JNK and total p38 levels showed no significant changes among groups, whereas p-JNK and p-p38 levels increased in a dose-dependent manner. Phosphorylation was quantified as p-JNK/JNK and p-p38/p38, and then normalized to the loading control (GAPDH). Data are presented as mean ± SD (*n* = 3). (A)Effects of curcumin on JNK/P38 signaling pathway protein expression in U2OS and MG63 cells (*x* ± *s*, *n* = 3). *P < 0.05 versus control group. (A-a) U2OS cells: Expression levels of P-JNK/JNK, P38, and P-P38/P38. *P < 0.05,**P < 0.01 and ***P < 0.001 versus control group. (A-b) MG63 cells: Expression levels of P-JNK/JNK, and P-P38/P38. *P < 0.05,***P < 0.001 versus control group.

To assess MAPK pathway involvement, Western blot analysis was performed for phosphorylated JNK mitogen-activated protein kinase (P-JNK) and phosphorylated p38 mitogen-activated protein kinase (P-p38) proteins. Curcumin treatment significantly increased P-P38 and P-JNK expression in a dose-dependent manner (**P < 0.01), while total P38 and JNK protein levels remained unchanged (*P* > 0.05; Figure 12).

### 3.8 TUNEL assay detection of late apoptotic features in osteosarcoma cells following treatment with different concentrations of curcumin

**Figure 13:**
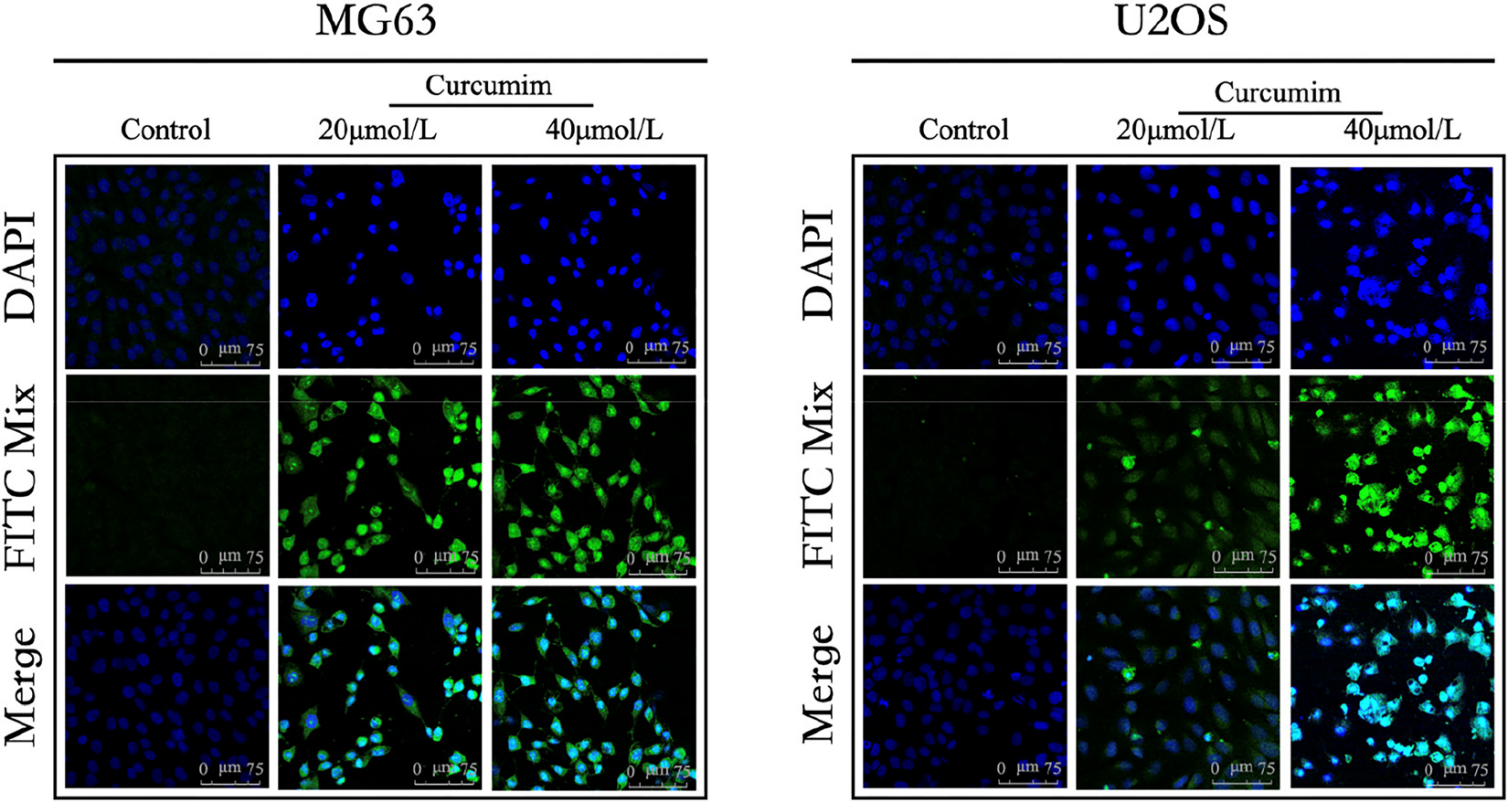
Apoptosis observed under fluorescence microscopy following treatment with different concentrations of curcumin. Curcumin significantly increased the proportion of TUNEL-positive cells in both MG63 and U2OS cells in a concentration-dependent manner, demonstrating that curcumin induces marked DNA fragmentation and apoptosis in osteosarcoma cells. The pro-apoptotic effect at 40 μmol/L was stronger than at 20 μmol/L, with consistent trends observed in both cell lines, providing direct morphological evidence supporting the conclusion that “curcumin inhibits osteosarcoma cell survival by inducing apoptosis.”

The TUNEL assay was employed to assess curcumin’s apoptotic effects on osteosarcoma cells (U2OS, MG63). Results demonstrated (Figure 13) that curcumin concentration-dependently increased the proportion of TUNEL-positive cells. Specifically, as curcumin concentration rose, DNA fragmentation in osteosarcoma cells markedly intensified, indicating curcumin promotes programmed cell death. Following treatment with low-dose curcumin (20 μmol/L), partial nuclear fragmentation and TUNEL-positive signals were observed in some cells. In contrast, high-dose treatment (40 μmol/L) resulted in extensive nuclear fragmentation and markedly enhanced TUNEL fluorescence, indicating a substantial increase in the proportion of late apoptotic cells.

## 4 Discussion

This study revealed the mechanism by which curcumin inhibits glycolysis and induces apoptosis in osteosarcoma cells by activating the p38 MAPK pathway through *in vitro* experiments and network pharmacology analysis. Compared with existing literature on curcumin’s effects in other tumors, the findings of this study exhibit both commonalities and specificities.

Across multiple solid tumors, curcumin has been reported to promote apoptosis by regulating energy metabolism and mitochondrial pathways. Its primary mechanisms include downregulating glycolysis-related enzymes, inhibiting ATP production, and reducing cellular energy supply. This is accompanied by Bax upregulation, Bcl-2 downregulation, and increased cleaved-PARP, thereby activating the mitochondrial-mediated apoptotic pathway. BCL-2 is widely recognized as an anti-apoptotic regulator and has been reported to be expressed across multiple tumor types, where it may influence cell survival and clinical behavior [14]. Aberrant BCL-2-associated phenotypes have also been documented in aggressive lymphoid malignancies and may relate to therapeutic responses [15]. Moreover, BCL-2 positivity has been observed in extranodal diffuse large B-cell lymphoma cases, further supporting the broad relevance of BCL-2 in tumor biology [16]. In this study, curcumin similarly exhibited glycolysis inhibition and mitochondrial apoptosis induction in osteosarcoma cells, suggesting that this “metabolism-signaling-apoptosis” coupling effect may be universal. Lactate dehydrogenase (LDH), particularly LDHA, is a key glycolytic node that catalyzes the conversion of pyruvate to lactate and regenerates NAD+, thereby sustaining high glycolytic flux in malignant cells. Therefore, changes in LDH/LDHA expression or lactate output are commonly used to reflect glycolytic dependency and the Warburg-type metabolic phenotype during antitumor interventions [17].

Unlike other tumors of epithelial origin, osteosarcoma cells exhibit greater dependence on glycolysis for cellular energy metabolism and are more sensitive to metabolic inhibition [18]. Our experiments revealed that curcumin significantly downregulated the expression of HK2, PKM2, and GLUT1, accompanied by a marked increase in P-JNK/P-P38 levels, while total JNK/P38 levels showed no significant change (Table 1). This indicates that curcumin primarily activates signaling events centered on the phosphorylation and activation of JNK/P38, rather than altering total protein levels. Previous studies have demonstrated that JNK/P38 MAPK plays dual pro-stress and pro-apoptotic roles in the biological processes of osteosarcoma, influencing tumor cell growth and invasion by regulating cell cycle progression, migration, and stress responses [19]. Our findings reveal that JNK/P38 activation is accompanied by glycolysis downregulation and enhanced apoptosis, suggesting a pro-apoptotic bias in osteosarcoma. Although phosphorylation of JNK/p38 correlates with reduced glycolytic markers and increased apoptosis, causality cannot be concluded without functional inhibition (pharmacological inhibitors or genetic knockdown/knockout) and rescue experiments.

**Table 1:** Summary of the effects of various concentrations of curcumin (0 μM, 20 μM, 40 μM) on OS cells, including key indicators such as cell morphology, scratch healing, expression of apoptosis-related proteins, glycolysis metabolism, and JNK/P38 signaling pathway.

Curcumin concentration	Cell morphology	Scratch healing rate	Apoptosis-related protein expression (WB)	Key enzymes of glycolysis (WB)	ATP	Glucose	Lactic acid	JNK/P38 activation
0 μmol/L	Normal spreading, tight connection	Fast healing	Bax decreased, BCL-2 increased, Cleaved-parp low	GLUT1, HK2, PKM2, and PDHA are highly expressed	High	Normal	Normal	p-JNK/p-p38 low
20 μmol/L	Decreased cell polarity and reduced pseudopodia	Significantly relieved, partially healed	Bax increased, BCL-2 decreased, and Cleaved-parp slightly increased	GLUT1, HK2, PKM2 decreased, PDHA slightly decreased	Decline	Decline	Decline	p-JNK/p-p38 upregulation
40 μmol/L	Shrinking, falling off, floating	Healing is significantly inhibited	Bax increased significantly, BCL-2 decreased significantly, and Cleaved-parp cleavage was enhanced	GLUT1, HK2, and PKM2 further decreased, and PDHA was significantly reduced	Significant decline	Significant decline	Significant decline	p-JNK/p-p38 were significantly upregulated

**Figure 14:**
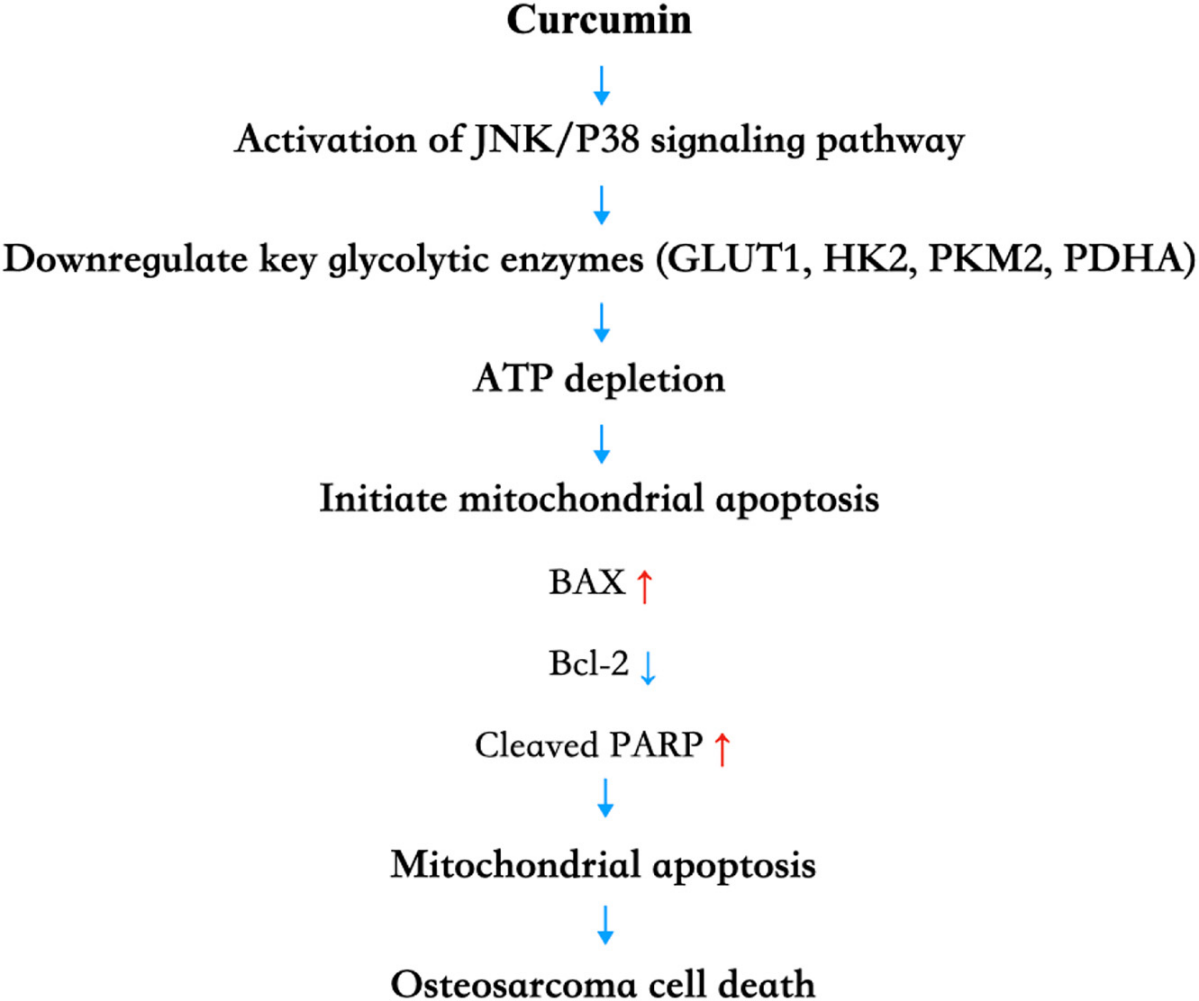
A mechanism model: “curcumin-JNK/P38 MAPK-glycolysis-mitochondrial apoptosis.

Unlike previous studies that examined curcumin’s effects from a single signaling or metabolic perspective, this research integrates the dynamic interplay among metabolism, signaling, and apoptosis at both experimental and network pharmacology levels. It proposes a mechanism model: “curcumin-JNK/P38 MAPK-glycolysis-mitochondrial apoptosis” (Figure 14). This model reveals the intrinsic link between curcumin-mediated programmed cell death in osteosarcoma cells through synergistic energy metabolism inhibition and signaling regulation. It provides a novel perspective on understanding curcumin’s antitumor mechanism in osteosarcoma and lays a theoretical foundation for future osteosarcoma treatment strategies based on metabolic-signaling cross-regulation.

Overall, curcumin primarily exerts its anti-osteosarcoma effects through the JNK/P38 MAPK branch within the MAPK family, regulating the balance between metabolism and apoptosis, rather than broadly influencing the entire MAPK axis. However, the causal role of the MAPK pathway has yet to be directly validated through loss-of-function experiments. To further clarify the pathway dependency of curcumin’s effects, subsequent studies may design the following experiments: MAPK pathway inhibitor intervention studies and downstream transcription factor detection:

1. Pharmacological inhibition of the MAPK pathway: While treating osteosarcoma cells with curcumin, co-administer a JNK-specific inhibitor (e.g., SP600125) or a p38 inhibitor (e.g., SB203580). Assess the following endpoints using CCK-8 assays, flow cytometry, and Western blotting:
  1.1 Whether the expression of glycolysis-related proteins (HK2, PKM2, GLUT1) is restored;
  1.2 Whether changes in apoptotic markers (Bax/Bcl-2 ratio, cleaved PARP) are reversed;
  1.3 Whether migratory capacity (wound-healing assay) and ATP production are partially recovered.
  If these inhibitors significantly abrogate the effects of curcumin, this would provide direct evidence that the JNK/p38 pathway is a central mediator of curcumin-induced glycolytic suppression and apoptosis.
2. Assessment of downstream transcription factor activity: The MAPK pathway can regulate downstream gene expression via phosphorylation of transcription factors such as c-Jun and ATF2. ChIP assays and/or luciferase reporter assays are recommended to determine whether curcumin, through activation of p-JNK and p-p38, enhances c-Jun/ATF2 binding and transcriptional activity at the promoters of glycolytic enzyme genes (e.g., HK2 and PKM2). If curcumin treatment increases c-Jun/ATF2 occupancy at these promoters, this would further clarify the transcriptional regulatory cascade linking MAPK activation to glycolytic inhibition.
  Collectively, these perturbation studies would address several key questions:
  2.1 Whether JNK and p38 function synergistically or independently in mediating curcumin’s effects;
  2.2 Whether the MAPK pathway directly regulates transcription of glycolytic enzyme genes, or instead induces apoptosis indirectly by disrupting cellular energy homeostasis.

This study confirms that curcumin inhibits glycolysis and induces apoptosis in osteosarcoma cells by activating the JNK/P38 MAPK signaling axis. Phosphorylated JNK and P38 expression increased following curcumin treatment; however, the direct causal relationship between these two factors and glycolysis inhibition/apoptosis induction requires validation through functional intervention experiments. Future studies may consider co-adding specific inhibitors of JNK or P38 (e.g., SP600125 or SB203580) to curcumin-treated osteosarcoma cells to observe their effects on key glycolytic protein expression and apoptosis rates (glucose uptake is critical for tumor cell survival [20], 21]). Should these inhibitors significantly reverse curcumin-induced glycolysis inhibition and apoptosis promotion, it would confirm the central role of the JNK/P38 signaling pathway in curcumin’s mechanism of action. Furthermore, elucidating the activity of downstream transcription factors in the MAPK pathway (e.g., c-Jun, ATF2) and their regulation of glycolysis-related genes will help establish a complete mechanistic chain from pathway activation to cellular metabolic reprogramming and fate determination.

Overall, our data indicate that curcumin suppresses glycolytic activity and induces apoptosis in osteosarcoma cells, accompanied by activation of the JNK/p38 MAPK branch. These findings support a model in which metabolic perturbation and stress-activated kinase signaling are coupled during curcumin-triggered cell death.

This study has several limitations. First, although JNK/p38 phosphorylation correlated with reduced glycolytic markers and enhanced apoptosis, causality cannot be established without functional inhibition and rescue experiments. Second, the current work is mainly based on *in vitro* assays in two osteosarcoma cell lines; additional validation in orthotopic or xenograft models is required to evaluate translational relevance. In future studies, we will apply pharmacological inhibitors (e.g., SP600125 for JNK and SB203580 for p38) and/or genetic silencing (siRNA/shRNA targeting MAPK8 and MAPK14) to test whether blocking JNK/p38 reverses curcumin-induced glycolytic suppression and apoptosis. We will also incorporate ROS modulation (e.g., N-acetylcysteine) and metabolic flux analyses (e.g., Seahorse ECAR measurements) to further delineate the upstream–downstream relationship among ROS, JNK/p38 activation, glycolytic reprogramming, and apoptotic execution.

This study has certain limitations. First, the causal role of the JNK/P38 MAPK pathway in mediating curcumin’s effects, though strongly suggested by our pharmacological and network pharmacology data, requires definitive validation through genetic loss-of-function experiments. Second, our conclusions are derived entirely from *in vitro* cell line models, which cannot fully replicate the complexity of the tumor microenvironment *in vivo*. The absence of *in vivo* validation or more advanced models limits the direct translational relevance of our current findings. Third, the experimental design did not include a non-tumorigenic osteoblast control or standard pharmacological positive controls, which would have strengthened the specificity and comparability of our results. Future studies designed to address these limitations are essential to solidify the proposed mechanism and evaluate the therapeutic potential of curcumin in osteosarcoma.

At the translational medicine level, although *in vitro* studies demonstrate curcumin’s potent anti-osteosarcoma activity, its inherent pharmacokinetic limitations – poor water solubility, low oral bioavailability, and rapid *in vivo* metabolism [22] – remain major obstacles to clinical application. To overcome these barriers, developing novel drug delivery systems (e.g., nanotargeted formulations based on liposomes or polymers) [23], [24], [25], [26] to enhance curcumin’s accumulation and stability within tumor tissues has become a key research focus. Concurrently, evaluating the combined efficacy of curcumin with conventional chemotherapeutic agents in more physiologically relevant animal models (such as patient-derived tumor xenograft models) is crucial for establishing its feasibility as an adjunctive treatment strategy for osteosarcoma.

## Supplementary Material

Supplementary Material
