# Supplementary material for: Curcumin induces apoptosis in osteosarcoma cells by regulating the glycolytic pathway via the MAPK axis: a mechanistic study
Source: Open Life Sci. 2026 Mar 10;21(1):20251296. doi: 10.1515/biol-2025-1296 (PMC12974748; doi:10.1515/biol-2025-1296)
Supplement: Supplementary file 1 — Supplementary Material [file j_biol-2025-1296_suppl_001.docx]

**Supplementary Table 1 **Details of reagents, antibodies, and assay kits used in this study****

| Experimental Reagents/Antibodies/Equipment | Catalog Number/Model Number | Dilution ratio | Manufacturer |
| --- | --- | --- | --- |
| U2OS and MG63 | Not applicable | Not applicable | Sigma Company, USA |
| U2OS and MG63 Specific Medium | G4540 and G4511 | Not applicable | Wuhan Seville Biotechnology Co., Ltd. |
| Curcumin | C408195 | Not applicable | Shanghai Aladdin Biochemical Technology Co., Ltd. |
| CCK-8 Assay Kit | C0038 | Not applicable | Shanghai Biyuntian Biotechnology Co., Ltd. |
| Annexin V-FITC Stain | C1062S | Not applicable | Shanghai Biyuntian Biotechnology Co., Ltd. |
| HRP-conjugated goat anti-rabbit secondary antibody | GB23303 | 1:10000 | Wuhan Seville Biotechnology Co., Ltd. |
| ECL Chemiluminescent Reagent | G2020-1  G2020-2 | 1:1 | Wuhan Seville Biotechnology Co., Ltd. |
| FITC staining solution | C1062S | Not applicable | Shanghai Biyuntian Biotechnology Co., Ltd. |
| Protein BCA Assay Kit | P0010 | Not applicable | Shanghai Biyuntian Biotechnology Co., Ltd. |
| Protein-Free Rapid Blocking Solution | G2052-500ML | Not applicable | Wuhan Seville Biotechnology Co., Ltd. |
| TUNEL Apoptosis Detection Kit | G1501-50T | Not applicable | Wuhan Seville Biotechnology Co., Ltd. |
| Bax | R22708 | 1:1000 | Chengdu Zhengneng Biotechnology Co., Ltd. |
| Bcl-2 | R23309 | 1:1000 | Chengdu Zhengneng Biotechnology Co., Ltd. |
| Cleaved-Parp | R09874 | 1:1000 | Chengdu Zhengneng Biotechnology Co., Ltd. |
| HK2 | P29803 | 1:1000 | Chengdu Zhengneng Biotechnology Co., Ltd. |
| PDHA | P29803 | 1:1000 | Chengdu Zhengneng Biotechnology Co., Ltd. |
| P-JNK | P5983 | 1:1000 | Chengdu Zhengneng Biotechnology Co., Ltd. |
| P-P38 | Q16539 | 1:1000 | Chengdu Zhengneng Biotechnology Co., Ltd. |
| PKM2 | P14618 | 1:1000 | Chengdu Zhengneng Biotechnology Co., Ltd. |
| GLUT1 | P11166 | 1:1000 | Chengdu Zhengneng Biotechnology Co., Ltd. |
| glyceraldehyde3phosphatedehydrogenase，GAPDH | SC47724 | 1:1000 | Santa Cruz Company, USA |
| P38 | 14064-1-AP | 1:1000 | Proteintech |
| JNK | 51153-1-AP | 1:1000 | Proteintech |
| Glucose (GOPOD Oxidase Method) Assay Kit | A-RXFG0164-48  B-RXFG0164-96 | A: B=2:1 | Quanzhou Ruixin Biotechnology Co., Ltd. |
| Quantitative Detection Kit for Mouse Ca-ATPase | RX2D235796 | Not applicable | Quanzhou Ruixin Biotechnology Co., Ltd. |
| L-Lactic Acid (LA) Colorimetric Assay Kit | E-BC-K044-M | Not applicable | Wuhan Ilarite Biotechnology Co., Ltd. |
| Enzyme-Linked Immunosorbent Assay Reader | Bole 550 | Not applicable | Shanghai Lingcheng Biotechnology Co., Ltd. |
| Multifunctional Fluorescence Microplate Reader | PHERAstar® FSX | Not applicable | BMG LABTECH GmbH, Germany |
| BD Vertical Electrophoresis System | Not applicable | Not applicable | Shanghai Lingcheng Biotechnology Co., Ltd. |
| Flow Cytometer | Not applicable | Not applicable | Shanghai Lingcheng Biotechnology Co., Ltd. |
